# Evaluation of the causal relationship between smoking and schizophrenia in East Asia

**DOI:** 10.1038/s41537-022-00281-5

**Published:** 2022-09-09

**Authors:** Mei-Hsin Su, Rou-Yi Lai, Yen-Feng Lin, Chia-Yen Chen, Yen-Chen A. Feng, Po-Chang Hsiao, Shi-Heng Wang

**Affiliations:** 1 Department of Occupational Safety and Health, College of Public Health, China Medical University, Taichung, Taiwan; 2 Department of Public Health, College of Public Health, China Medical University, Taichung, Taiwan; 3 Center for Neuropsychiatric Research, National Health Research Institutes, Miaoli, Taiwan; 4 Biogen, Cambridge, MA USA; 5 Stanley Center for Psychiatric Research, Broad Institute of MIT and Harvard, Cambridge, MA USA; 6 Institute of Epidemiology and Preventive Medicine, College of Public Health, National Taiwan University, Taipei, Taiwan

**Keywords:** Schizophrenia, Biomarkers

## Abstract

Cigarette smoking has been suggested to be associated with the risk of schizophrenia in observational studies. A significant causal effect of smoking on schizophrenia has been reported in European populations using the Mendelian randomization approach; however, no evidence of causality was found in participants from East Asia. Using Taiwan Biobank (TWBB), we conducted genome-wide association studies (GWAS) to identify susceptibility loci for smoking behaviors, including smoking initiation (*N* = 79,989) and the onset age (*N* = 15,582). We then meta-analyzed GWAS from TWBB and Biobank Japan (BBJ) with the total sample size of 245,425 for smoking initiation and 46,000 for onset age of smoking. The GWAS for schizophrenia was taken from the East Asia Psychiatric Genomics Consortium, which included 22,778 cases and 35,362 controls. We performed a two-sample Mendelian randomization to estimate the causality of smoking behaviors on schizophrenia in East Asia. In TWBB, we identified one locus that met genome-wide significance for onset age. In a meta-analysis of TWBB and BBJ, we identified two loci for smoking initiation. In Mendelian randomization, genetically predicted smoking initiation (odds ratio (OR) = 4.00, 95% confidence interval (CI) = 0.89–18.01, *P* = 0.071) and onset age (OR for a per-year increase = 0.96, 95% CI = 0.91–1.01, *P* = 0.098) were not significantly associated with schizophrenia; the direction of effect was consistent with European Ancestry samples, which had higher statistical power. These findings provide tentative evidence consistent with a causal role of smoking on the development of schizophrenia in East Asian populations.

## Introduction

Schizophrenia is often comorbid with smoking behaviors. Both case-control and cohort studies indicated that smokers were at a significantly higher risk of schizophrenia than non-smokers^1^, especially heavy-smokers^2^. Meta-analyses, including cohort studies, also indicated that smokers have a two-fold increased relative risk of schizophrenia than non-smokers^2,3^. In addition, the comorbidity of smoking and schizophrenia is associated with poorer prognosis^4^ and aggravated mental symptoms, such as flat affect, delusions, and hallucinations^5^.

Some possible explanations have been proposed for the association between smoking behaviors and schizophrenia. One argument is that substance use may lead to schizophrenia. According to reports, 90% of patients with schizophrenia started to smoke before the onset of their illness^5^, and adolescents with smoking behaviors were more likely to develop schizophrenia than non-smoking adolescents^1,6^. These studies used temporal sequence approach to infer the possibility that substance use may lead to schizophrenia. Another possible reason is shared genetic architecture. In our previous study, we calculated the polygenetic risk scores of schizophrenia based on genome-wide association studies (GWAS), and found that the score was positively associated with lifetime tobacco smoking^7^. On the other hand, the polygenic risk scores of cotinine concentrations also significantly predict schizophrenia diagnosis^8^. In addition, a genetic correlation was found between schizophrenia and regular smoking^9^. Self-medication is another hypothesis for the association between smoking and schizophrenia. Patients with schizophrenia may try to diminish their symptoms and the side effects of antipsychotic drugs through nicotine consumption, which can increase hepatic clearance and restore the dopamine blockade^10^. Animal models also provided evidence that chronic nicotine administration reversed hypofrontality by nicotinic acetylcholine receptor (nAChR) modulation and further attenuated the symptoms of schizophrenia^11,12^.

The association between smoking and schizophrenia has been established, but the causal relationship between them remains conflicting. Investigating the causal relationship between smoking and schizophrenia may help in blocking the path to schizophrenia and subsequently reduce the disease burden. Owing to the infeasibility of experimentally testing for effects of smoking on development of schizophrenia in humans, Mendelian randomization is an alternative study design for investigating causality using a genetic approach. Mendelian randomization adopts the concept of randomized control trials, and genetic variants can be used as instrumental variables for exposure of interest to uncover potential causal association. Mendelian randomization approach is not biased by reverse causation or confounding when assuming that genetic instruments are directly associated with the exposure, genetic instruments are not related to confounders, and no pleiotropy is allowed from genetic instruments to outcome. Previous Mendelian randomization studies have suggested that smoking shows causal effect on schizophrenia, and vice versa^13–16^. However, most studies were conducted in Caucasian populations with a sufficient sample size for GWASs for smoking and schizophrenia. Only one Mendelian randomization analysis from East Asian populations did not support such causality^17^. The negative finding may have resulted from insufficient power for the Mendelian randomization analysis with a limited sample size for GWAS. Therefore, estimating the causal relationship with a larger sample size in East Asian populations can help provide precise results.

Since smoking behaviors are associated with various physical^18,19^ and mental illnesses^2,7^, investigating the causality between them can help confirm the existence of causality between these two phenotypes, which can further identify the causal risk factors, which is preventable, for schizophrenia. In addition, in order to conduct a well-powered GWAS for smoking initiation and onset age among East Asian populations, we meta-analyzed the Taiwan Biobank (TWBB) and Biobank Japan (BBJ). We then examined the causal relationship between these two smoking behaviors and schizophrenia in East Asian populations.

## Results

### Smoking GWAS in East Asian populations

**Fig. 1:**
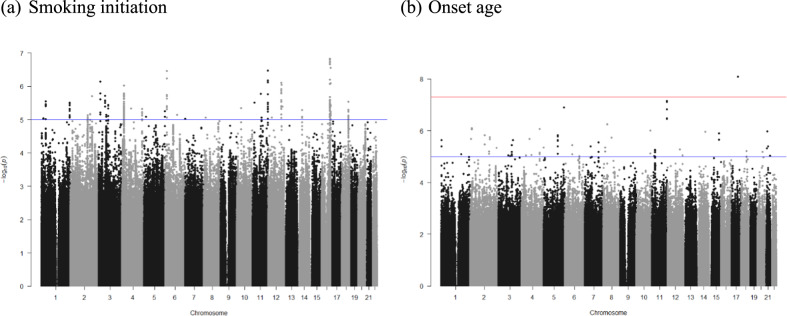
Manhattan plot for smoking behaviors among Taiwan Biobank data. (**a**) Manhattan plot of smoking initiation, (**b**) Manhattan plot of onset age. The vertical axis indicates the value of –log(*p*-value) for genome-wide association analysis, and the horizontal axis indicates chromosome number. The red line indicates the genome-wide significant level (*p* < 5E-08) and the blue line indicates suggest significant level (*p* < 1E-05).

Using individual genotyping and phenotyping data from the TWBB, we conducted GWAS to identify susceptibility loci for smoking behaviors, including smoking initiation (*N* = 79,989) and the onset age (*N* = 15,582). The Manhattan plot for two smoking traits is shown in Fig. 1, and one locus (rs553874586) was identified (Table 1). This single nucleotide polymorphism (SNP) in the *MMD* gene on chromosome 17 is associated with onset age (beta = −3.77, SE = 0.654, *P* = 8.08 × 10^−9^).

**Table 1:** Genome-wide association analysis for smoking behavior in East Asian populations.

Lead SNP	chr	Position (hg38)	Effect allele	gene	TWBB			BBJ			Combined		
					Beta	SE	*p*	Beta	SE	*p*	Beta	SE	*p*
Smoking initiation													
rs79574881	8	13420362	A	*DLC1*	−0.06	0.046	0.2039	−0.02	0.004	1.10E-07	−0.02	0.004	**2**.**84E-08**
rs2156008	18	55614013	A	*TCF4*	0.06	0.014	7.90E-06	0.01	0.002	5.30E-07	0.01	0.002	**3**.**21E-08**
Onset													
rs553874586	17	55405197	A	*MMD*	−3.77	0.654	**8**.**08E-09**	—	—	—	−3.77	0.654	**8**.**08E-09**

In the GWAS meta-analysis of TWBB and BBJ (*N* = 245,425 for smoking initiation and *N* = 46,000 for onset age of smoking), two SNPs met genome-wide significance for smoking initiation (Table 1): rs79574881 (at 8p22 with beta = −0.02, SE = 0.004, *P* = 2.84 × 10^−8^) and rs2156008 (at 18q21.2 with beta = 0.01, SE = 0.002, *P* = 3.21 × 10^−8^).

### Causal relationships of smoking and schizophrenia by Mendelian randomization study

Five Mendelian randomization methods were applied, including inverse variance-weighted (IVW), weighted median, weighted mode, Mendelian Randomization Pleiotropy Residual Sum and Outlier (MR-PRESSO), and MR Egger. We used 8 independent instrumental variables associated with smoking initiation at a lenient genome-wide significance threshold of *P* < 5 × 10^−7^, and 5 independent instrumental variables associated with onset age of *P* < 1 × 10^−6^ for Mendelian randomization analysis. The causal relationships of smoking behaviors and schizophrenia using Mendelian randomization analyses are displayed by scatter plots for the causal estimation (Supplementary Fig. 1) and forest plots for the effect of each instrumental variable (Supplementary Fig. 2). No heterogeneity was detected in the Mendelian randomization analysis for causality of the two smoking traits on schizophrenia (Supplementary Table 1).

The IVW method showed that genetically predicted smoking initiation was not significantly associated with an increased risk of schizophrenia (odds ratio [OR] = 4.00, 95% confidence interval [CI] = 0.89–18.01, *P* = 0.071) (Table 2). MR-PRESSO showed a significant causality of smoking initiation on schizophrenia (OR) = 4.00, *P* = 0.0122). Other MR methods provided similar estimates of OR around 4 but did not reach statistical significance. In addition, no overall pleiotropy was detected (*P*-value for MR Egger intercept = 0.99).

**Table 2:** The causal estimation of smoking behaviors on schizophrenia in East Asian populations.

Exposure	N SNP	Mendelian randomization method	N outliers	OR (95% CI)	*p*-value
Smoking initiation	8	IVW		4.00 (0.89–18.01)	0.0714
		Weighted median		3.44 (0.56–21.11)	0.1826
		Weighted mode		4.57 (0.30–69.27)	0.3091
		MR-PRESSO	0	4.00 (1.78–9.98)	0.0122
		MR Egger		4.14 (0.01–>99)	0.6562
		(intercept, SE)		−0.0003 (0.0281)	0.9906
Onset	5	IVW		0.96 (0.91–1.01)	0.0982
		Weighted median		0.97 (0.91–1.03)	0.5071
		Weighted mode		0.97 (0.90–1.05)	0.3311
		MR-PRESSO	0	0.96 (0.91–1.01)	0.1736
		MR Egger		0.93 (0.83–1.05)	0.3496
		(intercept, SE)		0.0170 (0.0367)	0.6757

For causality of onset age of smoking on schizophrenia, the IVW method showed that genetically predicted smoking onset was not significantly associated with schizophrenia (OR for a per-year increase in onset = 0.96, 95% CI = 0.91–1.01, *P* = 0.098) (Table 2). Other MR methods provided similar estimates. There was no overall pleiotropy according to the MR Egger intercept testing (*P*-value for MR Egger intercept = 0.68).

### Bidirectional Mendelian randomization

To ascertain the possible causal effect of schizophrenia on smoking behaviors, we also conducted bidirectional Mendelian randomization using East Asia data from Psychiatric Genomics Consortium (PGC) to identify genetic instruments for schizophrenia. There are 18 instrumental variables for schizophrenia (*P* < 5 × 10^−8^) included for bidirectional Mendelian randomization analysis. The findings did not support the causality of schizophrenia on the two smoking traits (see Supplementary Table 2).

### Genetic correlation of smoking and schizophrenia

No significant genetic correlation was found between smoking behavior and schizophrenia for East Asian populations (Supplementary Table 3). The genetic correlations were low, both for smoking initiation (*r*_g_ = −0.003, *P* = 0.94) and onset age (*r*_g_ = 0.102, *P* = 0.17) and schizophrenia.

## Discussion

Using improved-power GWAS data from East Asia populations, we detected three variants related to smoking initiation and onset age. The Mendelian randomization analysis provided little evidence of the harmful effect of smoking initiation and earlier age of smoking initiation on risk for schizophrenia in East Asia populations.

Concerning the linkage disequilibrium (LD), we only report the top significant SNP for each LD block. Three variants were detected in relation to smoking behaviors in a meta-analysis of East Asian populations. The top SNP is rs553874586, which is found to be associated with onset age of smoking, located on *the MMD* gene in chromosome 17. *MMD* gene is a protein-coding gene, and its molecular functions include protein kinase activity and signaling receptor activity. Other SNPs include rs79574881 and rs2156008 which are associated with smoking initiation. Rs79574881 is located on *the DLC1* gene, which encodes a GTPase-activating protein. It is a tumor suppressor gene and has been reported to be related to prostate, lung, and breast cancers^20,21^. Further, variants in *the DLC1* gene have also been associated with nicotine dependence^22^. Rs2156008 is located on *the TCF4* gene, which is a protein-coding gene related to nervous system development. Many SNPs in *the TCF4* gene are associated with schizophrenia or schizophrenia endophenotypes^23^. Moreover, the gene effects were modulated by smoking behavior, where heavy-smokers showed stronger gene effects on the schizophrenia endophenotype than light-smokers and non-smokers^24^.

Consistent and strong evidence has been reported for the causal effect of smoking initiation on schizophrenia among European-origin individuals^13–16^; however, this issue has been less discussed in East Asian populations. Only one Mendelian randomization analysis in East Asian participants with limited statistical power (sample size for smoking GWAS: 165,436 (BBJ); sample size for schizophrenia GWAS: 7,711 cases and 18,327 controls) reported no significant causal effect of smoking initiation on schizophrenia (OR = 0.32, *P* = 0.13)^17^. Concerning that if the causal effect is true, the biological mechanism should be presented even in different populations. Our study increased the sample size for exposure and outcome variables, however, only little evidence was observed for causality of smoking on schizophrenia. The direction of our causal effect estimate of smoking initiation on schizophrenia in East Asian populations (OR around 4) was in line with previous causal effect estimate among European-origin individuals (OR ranged 1.3–1.6)^13–15^, and the lack of significance of causality in our study may be due to the relatively insufficient sample size and power compared with previous Mendelian randomization study in European populations. In addition to Mendelian randomization, some other studies used a variety study designs and supported the possibility of causality. One retrospective study reported that 90% of patients with schizophrenia started smoking before the onset of their disease^5^, and longitudinal studies have pointed out that cigarette smoking increases the risk of developing schizophrenia and consistently reported a dose-response relationship of smoking quantity^1,25–27^.

Previous studies have provided evidence of shared polygenic risks between smoking and schizophrenia. For example, researchers found that the risk of schizophrenia increased with an increase in the polygenic risk score of plasma cotinine concentration, and a higher genetic score for schizophrenia increased the risk of smoking behaviors^8^. Some other studies reported a positive correlation between polygenic risk scores between regular smoking and schizophrenia^9^ and a significant association between polygenic risk score of smoking behaviors with schizophrenia^28^ to demonstrate the shared genetic liability between schizophrenia and smoking behaviors, which also describes the possible biological mechanism between smoking behaviors and schizophrenia.

The genetic correlation between smoking and schizophrenia did not achieve statistical significance in our study. Previous findings among European populations related to the same issue were inconsistent. A study reported small positive genetic correlation between smoking initiation and schizophrenia^13^ but others did not detect significance^16,29^. A study detected a small negative genetic correlation between onset age of smoking and schizophrenia^13^ but another did not^30^. This inconsistency may come from different sample size of GWAS summary data and a variety of prevalence of smoking behaviors in different populations.

In our Mendelian randomization analyses among East Asian populations, the sample size for smoking GWAS was insufficient to provide enough instruments that met genome-wide significance (*P* = 5 × 10^−8^). Instead, we used a lenient P-value threshold at *P* = 5 × 10^−7^ for smoking initiation and *P* = 1 × 10^−6^ for onset age to select instrumental variables (n = 8 and 5 for smoking initiation and onset age, respectively). In addition, we also used a more lenient threshold (*P* = 1 × 10^−5^) to include more instrumental variables, and the results for Mendelian randomization analyses still did not reach statistical significance (data not shown). Relaxing threshold for selecting instrumental variables may recruit weak instrumental variables and lead to bias in Mendelian randomization analysis. Furthermore, we only included two smoking behaviors (smoking initiation and onset age of smoking) in the analysis; hence, the causal effect of a wide range of smoking traits on schizophrenia needs to be examined further.

In conclusion, this study identified two SNPs related to smoking initiation and one SNP related to the onset age. Though the causal effect of smoking on schizophrenia in these East Asian samples did not yield significant results, the direction of effect was consistent with European Ancestry samples, which had the benefit of higher statistical power. Thus, these findings provide tentative evidence consistent with a causal role of smoking on the development of schizophrenia in East Asian populations. Future efforts to elucidate the mechanisms underlying the association between smoking and schizophrenia are needed and may help early prevention.

## Methods

### GWAS for smoking

This study used individual genotyping and phenotyping data from TWBB, the largest government-supported biobank in Taiwan since 2012. TWBB recruits community-based samples aged 30–70 years who are cancer-free at recruitment. The internal review board approved the recruitment and data collection procedures of TWBB. Each participant signed an approved informed consent form, provided blood samples, and underwent physical examinations and face-to-face interviews. This study was approved by the Central Regional Research Ethics Committee of China Medical University, Taichung, Taiwan (CRREC-108-30).

Genotyping of 95,238 TWBB participants was performed using customized TWBB chips and processed on the Axiom Genome-Wide Array Plate System (Affymetrix, Santa Clara, CA, USA). Furthermore, 26,274 participants were genotyped on the TWBv1 chip, and 68,964 participants were genotyped on the TWBv2 chip. We conducted quality control and imputation of the two chips separately. Quality control included the exclusion criteria of variants with call rate <95%, individuals with more than 5% missing variants, minor allele frequency (MAF) < 0.001, and deviation from Hardy-Weinberg equilibrium with *P* < 1 × 10^−6^. Imputation was performed based on the 973 TWBB panels from whole-genome sequencing in TWBB participants and 504 East Asia panels in the 1000 Genomes project, variants with MAF ≥ 0.5%, and imputation INFO score ≥0.7. In addition, cryptic relatedness was removed, and we estimated identity by descent (IBD) sharing coefficients, PI-HAT = probability (IBD = 2) + 0.5 × probability (IBD = 1), between any two participants in KING and excluded one individual from a pair with PI-HAT ≥ 0.1875.

GWAS for two smoking behaviors included smoking initiation (yes/no) (*N* = 79,989) and onset age (*N* = 15,582) in TWBB. We performed linear or logistic regression in PLINK for association tests with adjustment for age, age^2^, sex, age by sex interaction, age^2^ by sex interaction, and the top 20 principal components. In addition, we performed a genetic association test separately for the two chips and performed an inverse-variance-weighted fixed-effect meta-analysis in METAL. For each trait, the sample size and heritability estimates for each chip and genetic correlation estimates^29,31^ between the two chips are detailed in Supplementary Table 4.

To maximize the power of genetic discovery in East Asia, we meta-analyzed GWAS from TWBB and BBJ. Genotyping for BBJ was carried out using the Illumina Human Omni Express Exome or the Human Exome platforms. In the quality control processes, samples with call rate <0.98, closely related individuals, and outlier of the East Asian cluster were excluded. As for genetic variants, SNPs with MAF < 0.005 and call rate < 0.99 were removed. Imputation was performed based on 1000 Genome (phase1v3). Only imputation quality score (*r*^2^) ≥ 0.7 were used for analysis. GWAS summaries for smoking initiation (*N* = 165,436) and for onset age (*N* = 30,418) were retrieved.

After meta-analyzed GWAS from TWBB and BBJ, the sample size was 245,425 for smoking initiation and 46,000 for onset age. The genetic correlation between TWBB and BBJ was median to high for smoking initiation but not for onset age (Supplementary Table 5).

### GWAS summary for schizophrenia

The GWAS summary of schizophrenia from the East Asia Psychiatric Genomics Consortium (PGC), including 22,778 cases and 35,362 controls^32^ from East Asia, and the diagnosis of schizophrenia was based on the Diagnostic Manual of Mental Disorders-IV (DSM-IV) system.

### Mendelian randomization

#### Genetic instrument for causality of smoking on schizophrenia

We mapped the variants from exposure (smoking phenotypes) data to outcome (schizophrenia) GWAS and preserved those that could be mapped to both. LD clumping was conducted based on *r*^2^ > 0.0001 within a 1,000 kb window to select independent variants. To recruit enough instrumental variables (at least five), we used a lenient threshold for SNP selection and identified eight SNPs for smoking initiation with *a P*-value of 5 × 10^−7^ and five SNPs for onset age with *a P*-value of 1 × 10^−6^. In order to recruit more genetic instruments, we also used a more lenient threshold at *P* < 1 × 10^−5^ which has 67 instrumental variables for smoking initiation and 27 instrumental variables for onset age of smoking.

#### Genetic instruments for bidirectional Mendelian randomization

A bidirectional Mendelian randomization was conducted to estimate the causal effect of schizophrenia on smoking behaviors and examine whether a Mendelian randomization study supports the argument of self-medication in patients with schizophrenia. The data for bidirectional Mendelian randomization were taken from GWAS summary data of East Asian populations with 18 independent SNPs of genome-wide significance (*P* < 5 × 10^−8^) for both smoking initiation and onset age.

#### Mendelian randomization analysis

In this study, five Mendelian randomization methods were applied as follows: inverse variance-weighted (IVW)^33,34^, weighted median^35^, weighted mode^36^, Mendelian Randomization Pleiotropy Residual Sum and Outlier (MR-PRESSO)^37^, and MR Egger^38^. IVW provides the best unbiased estimation in the absence of pleiotropy and under valid SNPs; hence, the main results for Mendelian randomization analysis are based on IVW method and the other four Mendelian randomization methods for the sensitivity analyses. We also examined pleiotropy using Egger intercept and heterogeneity using Cochran’s Q and Rücker’s Q.

### Genetic correlation

Genetic correlation between two smoking traits and schizophrenia in East Asians was conducted to detect whether common genetic variants existed between these two phenotypes. The genetic correlation was measured using LD score regression^29,31^.

## Supplementary information

SUPPLEMENTAL MATERIAL

## Data Availability

GWAS summary results for schizophrenia are available on the PGC website https://www.med.unc.edu/pgc/. GWAS summary results for smoking behaviors are available on the website of Biobank Japan http://jenger.riken.jp/en/result.
