# Supplementary material for: Evaluation of the causal relationship between smoking and schizophrenia in East Asia
Source: Schizophrenia (Heidelb). 2022 Sep 9;8(1):72. doi: 10.1038/s41537-022-00281-5 (PMC9463183; doi:10.1038/s41537-022-00281-5)
Supplement: Supplementary file 1 — SUPPLEMENTAL MATERIAL [file 41537_2022_281_MOESM1_ESM.docx]

**Supplementary**

**Supplementary Table**

**SUPP Table 1. Heterogeneity testing for Mendelian randomization**

**SUPP Table 2. Bidirectional Mendelian randomization of schizophrenia on smoking behaviors in East Asian populations**

**SUPP Table 3. Genetic correlation of smoking behaviors and schizophrenia among East Asian populations**

**SUPP Table 4. Genetic correlation of smoking behaviors between TWBB with different genotyping arrays**

**SUPP Table 5. Genetic correlation of smoking behaviors between TWBB and BBJ**

**Supplementary Figure**

**SUPP Figure 1. Scatter plot of the causal estimation for smoking behaviors on schizophrenia of East Asian populations**

**SUPP Figure 2. Forest plot of the causal estimation for smoking behaviors on schizophrenia of East Asian populations**

SUPP Table 1. Heterogeneity testing for Mendelian randomization

| Exposure phenotypes | Outcome | IVW | |  | MR-Egger | |
| --- | --- | --- | --- | --- | --- | --- |
|  |  | Cochran’s Q | *p* |  | Rücker's Q | *p* |
| Smoking initiation | Schizophrenia Asia | 2.0248 | 0.9585 |  | 2.0246 | 0.9174 |
| Onset | Schizophrenia Asia | 4.3808 | 0.3569 |  | 4.0901 | 0.2519 |
| Schizophrenia Asia | Smoking initiation | 40.3592 | 0.0012 |  | 40.0078 | 0.0008 |
| Schizophrenia Asia | Onset | 18.7435 | 0.3435 |  | 14.7529 | 0.5428 |

SUPP Table 2. Bidirectional Mendelian randomization of schizophrenia on smoking behaviors in East Asian populations.

| Outcome | N SNP | Mendelian randomization Method | N outliers | Beta (SE) | p-value |
| --- | --- | --- | --- | --- | --- |
| Smoking initiation | 18 | IVW |  | -0.0024 (0.0035) | 0.5012 |
|  | 18 | Weighted median |  | -0.0020 (0.0033) | 0.5328 |
|  | 18 | Weighted mode |  | -0.0015 (0.0035) | 0.6742 |
|  | 18 | MR-PRESSO | 1 | -0.0013 (0.0031) | 0.6823 |
|  | 18 | MR Egger |  | 0.0050 (0.0200) | 0.8058 |
|  |  | (intercept, SE) |  | -0.0008 (0.0020) | 0.7127 |
| Onset | 18 | IVW |  | -0.0027 (0.0028) | 0.3347 |
|  | 18 | Weighted median |  | -0.0028 (0.0031) | 0.3701 |
|  | 18 | Weighted mode |  | -0.0030 (0.0073) | 0.6852 |
|  | 18 | MR-PRESSO | 0 | -0.0027 (0.0028) | 0.3482 |
|  | 18 | MR Egger |  | 0.0513 (0.0271) | 0.0772 |
|  |  | (intercept, SE) |  | -0.0045 (0.0023) | 0.0630 |

SUPP Table 3. Genetic correlation of smoking behaviors and schizophrenia among East Asian populations.

| Trait | Smoking behaviors | | | | | |  | Schizophrenia | | | | | |  | Genetic correlation | | |
| --- | --- | --- | --- | --- | --- | --- | --- | --- | --- | --- | --- | --- | --- | --- | --- | --- | --- |
|  | Sample size | h*^2^_g_* (SE) | λ_GC_ | Mean *χ*^2^ | Intercept (SE) | Ratio |  | Sample size | h*^2^_g_* (SE) | λ_GC_ | Mean *χ*^2^ | Intercept (SE) | Ratio |  | r_g_ | SE | P-value |
| Smoking initiation | 245425 | 0.032  (0.002) | 1.165 | 1.175 | 1.012  (0.006) | 0.073 |  | 58140 | 0.246  (0.014) | 1.227 | 1.290 | 0.991 (0.008) | < 0 |  | -0.003 | 0.034 | 0.938 |
| Onset | 46000 | 0.030 (0.008) | 1.050 | 1.038 | 1.009 (0.006) | 0.230 |  |  |  |  |  |  |  |  | 0.102 | 0.074 | 0.171 |

SUPP Table 4. Genetic correlation of smoking behaviors between TWBB with different genotyping arrays

| Trait | TWBB batch 1 (TWBB2.0) | | | | |  | TWBB batch 2 (TWBB1.0) | | | | |  | Genetic correlation | | |
| --- | --- | --- | --- | --- | --- | --- | --- | --- | --- | --- | --- | --- | --- | --- | --- |
|  | h*^2^_g_* (SE) | λ_GC_ | Mean *χ*^2^ | Intercept (SE) | Ratio (SE) |  | h*^2^_g_* (SE) | λ_GC_ | Mean *χ*^2^ | Intercept (SE) | Ratio (SE) |  | r_g_ | SE | P-value |
| Smoking initiation | 0.07 (0.01) | 1.065 | 1.074 | 0.99 (0.01) | < 0 |  | 0.08 (0.02) | 1.032 | 1.036 | 1.00 (0.01) | < 0 |  | 0.781 | 0.140 | 2.46E-08 |
| Onset | 0.15 (0.04) | 1.034 | 1.026 | 0.99 (0.01) | < 0 |  | -0.02 (0.08) | 1.004 | 1.005 | 1.01 (0.01) | 1.59 (1.45) |  | NA | NA | NA |

SUPP Table 5. Genetic correlation of smoking behaviors between TWBB and BBJ

| Trait | TWBB (metal) | | | | | |  | BBJ | | | | | |  | Genetic correlation | | |
| --- | --- | --- | --- | --- | --- | --- | --- | --- | --- | --- | --- | --- | --- | --- | --- | --- | --- |
|  | Sample size | h*^2^_g_* (SE) | λ_GC_ | Mean *χ*^2^ | Intercept (SE) | Ratio |  | Sample size | h*^2^_g_* (SE) | λ_GC_ | Mean *χ*^2^ | Intercept (SE) | Ratio |  | r_g_ | SE | P-value |
| Smoking initiation | 79989 | 0.06 (0.01) | 1.093 | 1.101 | 1.00 (0.01) | < 0 |  | 165436 | 0.05 (0.002) | 1.201 | 1.193 | 1.01 (0.01) | 0.08 (0.04) |  | 0.746 | 0.077 | 1.82E-22 |
| Onset | 15582 | 0.06 (0.03) | 1.022 | 1.013 | 0.99 (0.01) | < 0 |  | 30418 | 0.04 (0.02) | 1.047 | 1.037 | 1.01 (0.01) | 0.26 (0.23) |  | 0.005 | 0.294 | 9.88E-01 |

SUPP Figure 1. Scatter plot of the causal estimation for smoking behaviors on schizophrenia of East Asian populations.

| 1. Smoking initiation   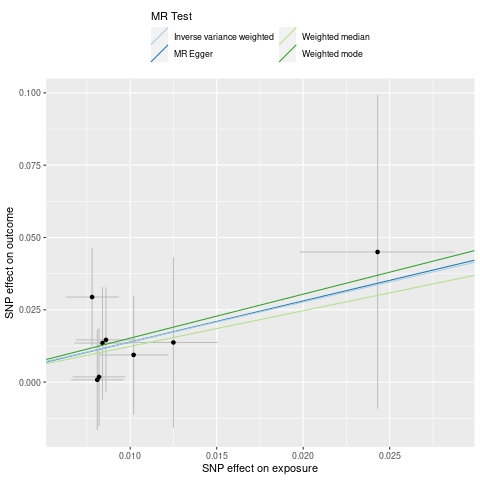 | 1. Onset   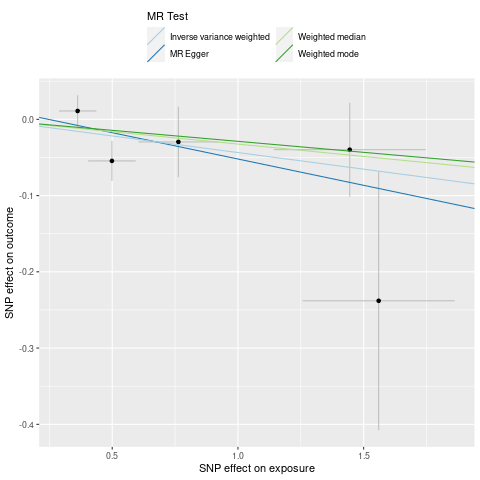 |
| --- | --- |
| 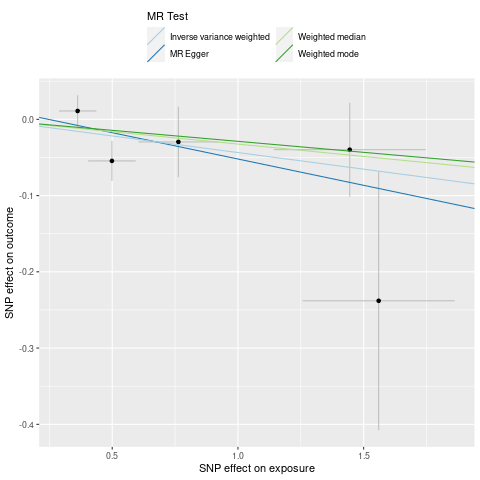 | |

SUPP Figure 2. Forest plot of the causal estimation for smoking behaviors on schizophrenia of East Asian populations.

| 1. Smoking initiation   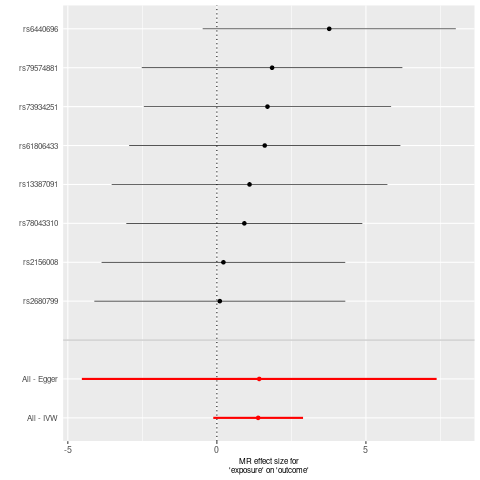 | 1. Onset   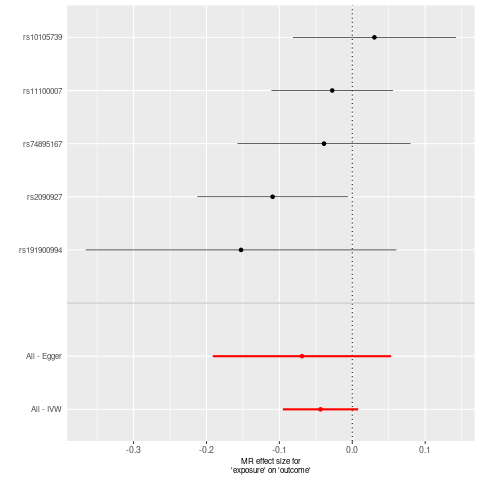 |
| --- | --- |
